# Trends and Factors Associated with Under-5 Mortality in Northwest Nigeria (2008–2018)

**DOI:** 10.5334/aogh.3564

**Published:** 2022-07-05

**Authors:** Osita K. Ezeh, Anastasia O. Odumegwu, Gladys H. Oforkansi, Uchechukwu D. Abada, Felix A. Ogbo, Piwuna C. Goson, Tanko Ishaya, Kingsley E. Agho

**Affiliations:** 1 School of Science and Health, University of Western Sydney, Locked Bag 1797, Penrith, NSW 2571, AU; 2 Anambra State Ministry of Health, Awka, NG; 3 Department of Economics, Nnamdi Azikiwe University, Awka, NG; 4 Department of Banking and Finance, Madonna University, Okija Campus, Anambra State, NG; 5 Translational Health Research Institute (THRI), Western Sydney University, Campbelltown Campus, Penrith NSW 2571, AU; 6 Barmera Medical Clinic [Lake Bonney Private Medical Clinic], 24 Hawdon Street, Barmera SA 5345, AU; 7 Department of Psychiatry, College of Health Sciences, University of Jos, Jos 930003, NG; 8 Department of Computer Science, University of Jos, Jos 930003, NG

**Keywords:** under-5 mortality, northwest, Nigeria, mortality rate, child mortality, geopolitical zone

## Abstract

**Background:**

The Nigeria Demographic and Health Survey (NDHS) revealed that the under-five mortality rate (U5MR) in the northwest geopolitical zone (NWGZ), Nigeria, increased by 1.1% from 185 to 187 deaths per 1,000 live births between 2013 and 2018, indicating a setback to the previously reported modest improvement in U5MR.

**Objectives:**

This study sought to examine trends and factors related to under-5 mortality (U5M) in NWGZ from 2008 to 2018.

**Methods:**

A combined NWGZ dataset extracted from the 2008, 2013 and 2018 NDHSs, with a sample of 32,015 singleton live births, including 3,745 under-5 deaths, was used. The U5MRs for each survey year and potential independent factors were obtained using the STATA “syncrmrates” command, and then the trends were examined. A logistic regression generalised linear latent and mixed model was used to explore the potential factors associated with U5M in NWGZ.

**Findings:**

In NWGZ, the U5MR declined by only 8.2% (from 195 to 179 per 1,000 live births between 2008 and 2018, respectively), with a similar trend observed among its seven states. Multivariable analyses indicated that maternal education (no formal or primary education), maternal non-use of contraception, a mother’s perception of the baby being small or very small, birth order (second to fourth or higher) with a shorter birth interval (≤2 years), younger or older maternal age (<20 years or ≥40 years old) and rural residence were significantly associated with U5M in NWGZ.

**Conclusion:**

Interventional initiatives including educating mothers on the benefits of contraceptive use, child spacing, kangaroo mother care of small-sized babies and promoting regular check-ups for older mothers will substantially reduce U5M in NWGZ.

## Background

Uneven variations in under-5 mortality (U5M) or deaths of children aged between 0 and 59 months in regional or geopolitical locations remain a substantial public health challenge in sub-Saharan Africa, including Nigeria. In a recent global child mortality estimate, Nigeria ranked the highest with the absolute number (0.858 million) of deaths among children under five years of age in 2019 [1], with a huge number occurring in the northwest geopolitical zone (NWGZ) of the country. Sadly, many of these deaths were preventable or treatable diseases (e.g., diarrhoeal diseases, respiratory tract infection and malaria) [2,3] with affordable and cost-effective interventions such as access to clean water and sanitation, use of mosquitoes’ treated bed net and prompt access to affordable and reliable medical care.

The NWGZ is one of the six geopolitical zones in Nigeria, comprising seven states (Jigawa, Kaduna, Kano, Katsina, Kebbi, Sokoto and Zamfara). The NWGZ continues to report one of the highest under-5 mortality rates (U5MR), with sharp variations among the different states. An example of this position can be observed in the 2008 Nigeria Demographic and Health Survey (NDHS), where NWGZ reported a high U5MR of 217 deaths per 1,000 live births [4]; this rate slightly decreased to 185 in the 2013 NDHS [5] and unexpectedly rose to 187 in the 2018 NDHS [6]. The current U5MR of 187 deaths per 1,000 live births implies that approximately one in every five children aged under five dies in NWGZ before reaching their fifth birthday, which is nearly four times the average rate reported for the southwest geopolitical zone (62 deaths per 1,000 live births) [6]. The NWGZ high U5MR is concerning and requires detailed investigation, given the successfully established and implemented government interventional initiatives in NWGZ states—especially Jigawa, Katsina, and Zamfara, in the past two decades. For example, the Partnership for Reviving Routine Immunization in Northern Nigeria (PRRINN) was established in 2006, whose core goals included decreasing the number of deaths among children below five years of age [7]. In 2008, PRRINN was expanded to incorporate maternal, newborn, and child health to enhance maternal and child survival [7]. Additionally, an initiative for health programmes for women was launched in 2012, whose primary aim was to save the lives of newborns and women, particularly in rural communities that lack skilled midwives [8].

Most population-based studies on U5M in Nigeria have focused on aggregated national data. These studies showed a wide variety of factors associated with U5M such as community (rural residence) [9], socioeconomic (mothers who had no formal education and exhibited poor or middle household wealth index) [9] and proximate (male gender, mother’s perceived newborn size at birth, caesarean delivery, birth order, birth interval [9], unskilled birth attendance, use of biomass/unclean fuel for cooking [10] and maternal non-use of modern family planning [11]. However, the literature is limited to disaggregated geopolitical zone and/or state-level estimates, especially in the NWGZ. In addition, national data can mask regional specific health and socioeconomic issues, with subsequent adverse impacts on policy and programmes implementation. Sub-national studies can reveal potential multi-layered cohesive structural and contextual factors that differentially impact interventional policies across geopolitical zones and/or within communities [12]. Information regarding changes over time in U5M across geopolitical zones and/or states is also lacking in the literature. This information is crucial for appraising the effectiveness of past or current early child health interventional coverage to guide new or refinement of present or future programmes.

To the author’s knowledge, no published population-based studies have investigated the trends and factors associated with U5M in the NWGZ of Nigeria. As a result, we examined U5MR trends in this study and investigated the potential characteristics associated with singleton U5M, using the NWGZ populations extracted from the Nigeria Demographic and Health Survey (NDHS) dataset between 2008 and 2018. Findings from this disaggregated NWGZ population with similar characteristics (e.g. religion, culture and socioeconomic activities) will guide health policymakers in formulating cost-effective, zone-specific intervention programmes to reduce U5M across the seven states in NWGZ.

## Methods

In this study, the NWGZ birth recodes file dataset (NGBR7AFL.DTA) extracted from 2008, 2013 and 2018 NDHSs was used. Information concerning live births and deaths was documented for women aged between 15 and 49 years who participated in the surveys. During the 10-year study period, the weighted total number of live births reported in NWGZ was 32,015, including 8,529 from the 2008 survey, 11,442 from the 2013 survey and 12,045 from the 2018 survey. These data were obtained using a multi-stage, stratified, cluster random sampling technique detailed elsewhere [4,5,6].

### Study-dependent variable

The dependent variable was U5M, which is defined as the death of a live-born singleton between birth and 59 months of life. The dependent variable took a binary form such that child death was referred to as a “case” (1 = if child death occurred in the study-specified age period) or “non-case” (0 = if a child was alive in the study-specified age period).

### Potential independent variables

The well-thought-out independent variables used in the analysis were influenced by Mosley and Chen’s framework of factors influencing child survival in developing countries [13] and other studies conducted on childhood mortality in Nigeria [9,10,11,14,15,16,17]. The potential independent variables comprised geographic location of residence (rural-urban residence) and state of origin categorised as Jigawa, Kaduna, Kano, Katsina, Kebbi, Sokoto and Zamfara, were tailored to the information available in the study’s combined dataset. Household economic status, individual-level factors and health service-related factors were also incorporated in the study analysis.

Maternal individual-level characteristics comprised education, literacy level, age, body mass index, occupation, and desire for pregnancy, while child characteristics comprised sex, mother’s perceived baby size at birth, birth order and birth interval. We also included paternal individual-level characteristics, such as education, number of women (or wives) in the household and occupation.

Health service-related factors, such as delivery assistance, mode of delivery, contraceptive use and place of birth, classified into home and health facilities were examined. Other important health service-related factors, such as antenatal and postnatal care services, were not assessed because only singleton live births at the five-year time point before the survey interview date were considered. Data concerning antenatal and postnatal care services in the 2008, 2013 and 2018 NDHSs were only documented for a mother’s last childbirth (or most recent birth) in the five years preceding the survey.

### Statistical analysis

Initially, the frequency tabulation of all potential factors of child survival was examined discretely for each survey year. The U5MRs for singleton live births for the specified age period were estimated using the “syncmrates” command in STATA, as described by Rutstein and Rojas [18]. Then, the crude odds ratios (OR) and adjusted odds ratios (AOR), which independently measured the magnitude of the impact of each factor associated with the study-dependent variable, were investigated by univariable and multivariable analyses, respectively, using a logistic regression generalised linear latent and mixed model (GLLAMM). Both univariable and multivariable analyses were carried out using STATA/MP, V.13 (StataCorp).

A multivariable logistic regression (GLLAMM) analysis was conducted using a manually stepwise backward elimination method, consistent with the literature [19,20]. This was used to identify independent factors that were significantly related to the study-dependent variable. The following criteria were considered to reduce the statistical bias during the manually backward elimination procedure: (1) estimated potential variables in the univariable model with a p-value < 0.25 were incorporated in the baseline multivariable model for the manual backward elimination procedure; (2) to double-check the manual backward elimination process, all potential independent variables were re-entered into the baseline multivariable model and re-examined; and (3) we tested and reported any potential variables with collinearity in the final model. The adjusted potential variables related to the study-dependent variable at a 0.05 significance level were retained and reported in the final model.

## Results

### Frequency of under-5 deaths (singleton) by study factors

A weighted total of 3,745 singleton live-born children below five years of age died in NWGZ between 2008 and 2018, which involved 505 (Jigawa), 485 (Kaduna), 942 (Kano), 578 (Katsina), 395 (Kebbi), 385 (Sokoto), and 454 (Zamfara) children. The proportion of children whose mothers had no schooling barely changed from 81% in 2008 to 80% in 2018, and that of children from poor households slightly decreased from 49% in 2008 to 38% in 2018 (Table 1).

**Table 1:** Distribution of and under-five mortality rate (U5MR) for each category of individual, household and community variables in Northwest geopolitical zone, Nigeria, 2008-2018.


CHARACTERISTIC	2008		2013		2018	
		
	N (%)	U5MR (95% CI)*	N (%)	U5MR (95% CI)*	N (%)	U5MR (95% CI)*

**Residence type**						

Urban	140 (12)	167 (152—182)	169 (13)	148 (136—160)	318 (19)	169 (155—184)

Rural	1050 (88)	195 (184—206)	1121 (87)	186 (177—196)	1345 (81)	191 (178—204)

**Northwest state**						

Jigawa	129 (11)	204 (189—219)	214 (17)	162 (145—178)	217 (13)	190 (175—205)

Kaduna	139 (12)	187 (164—210)	65 (5)	162 (143—181)	364 (22)	174 (158—191)

Kano	396 (33)	183 (165—200)	307 (24)	167 (153—182)	319 (19)	188 (173—203)

Katsina	202 (17)	191 (175—207)	154 (12)	160 (148—172)	287 (17)	185 (171—200)

Kebbi	71 (6)	205 (186—224)	152 (12)	161 (147—175)	219 (13)	187 (171—203)

Sokoto	157 (13)	199 (182—216)	145 (11)	159 (146—173)	125 (8)	193 (177—209)

Zamfara	96 (8)	199 (181—216)	253 (20)	152 (139—164)	132 (8)	191 (174—207)

**Household wealth index+**						

Rich	151 (13)	165 (150—181)	171 (13)	145 (135—154)	253 (15)	170 (155—185)

Middle	457 (38)	195 (181—209)	536 (42)	177 (161—193)	772 (46)	186 (170—201)

Poor	582 (49)	200 (187—212)	583 (45)	178 (166—191)	637 (38)	195 (182—207)

**Mother’s education**						

Secondary or higher	70 (6)	170 (154—186)	59 (5)	142 (129—155)	123 (7)	176 (160—193)

Primary	161 (13)	188 (173—204)	117 (9)	158 (144—171)	205 (12)	183 (164—202)

No education	959 (81)	196 (185—207)	1113 (86)	187 (174—200)	1335 (80)	189 (177—201)

**Mother’s literacy level**~						

Able to read	148 (12)	166 (147—185)	107 (8)	147 (134—159)	219 (13)	172 (156—188)

Cannot read	1025 (86)	194 (181—206)	1173 (91)	186 (174—197)	1444 (87)	187 (177—199)

**Mother’s age**						

< 20	86 (7)	209 (190—229)	95 (7)	157 (142—172)	106 (6)	193 (175—211)

20—29	534 (45)	184 (171—195)	586 (46)	172 (159—185)	817 (49)	179 (166—193)

30—39	411 (35)	183 (171—195)	430 (33)	168 (155—180)	540 (33)	184 (172—197)

40—49	159 (13)	198 (182—214)	178 (14)	159 (145—173)	201 (12)	188 (175—201)

**Mother’s body mass index~**						

Normal	790 (67)	186 (174—198)	984 (77)	175 (164—186)	427 (77)	186 (173—199)

Underweight	178 (15)	196 (181—211)	128 (10)	156 (144—169)	61 (11)	186 (167—203)

Overweight	148 (12)	190 (173—206)	119 (9)	158 (143—172)	54 (9)	184 (166—202)

Obese	65 (6)	198 (178—218)	49 (4)	151 (138—164)	13 (2)	187 (172—201)

**Wanted pregnancy**~						

Wanted then	1039 (87)	180 (168—191)	1225(95)	177 (166—188)	1604 (97)	176 (165—187)

Wanted later	39 (3)	200 (183—218)	13 (1)	152 (139—166)	45 (3)	188 (170—206)

Wanted no more	13 (1)	204 (184—224)	5 (0.4)	152 (136—167)	14 (1)	189 (169—209)

**Father’s education**~						

No education	767 (65)	200 (186—213)	893 (69)	188 (177—199)	1063 (64)	191 (180—203)

Primary	186 (16)	195 (181—209)	186 (14)	160 (150—171)	227 (14)	186 (172—200)

Secondary or higher	206 (17)	164 (149—179)	182 (14)	146 (134—157)	291 (18)	172 (157—187)

**Mother’s perceived baby size**~						

Average or larger	875 (74)	172 (160—183)	926 (72)	169 (159—179)	1268 (76)	171 (160—183)

Small or very small	222 (19)	213 (199—228)	300 (24)	173 (158—187)	389 (23)	196 (182—209)

**Sex**						

Female	551 (46)	183 (167—199)	588 (46)	173 (157—189)	831 (50)	178 (164—191)

Male	640 (54)	187 (174—200)	702 (54)	172 (161—183)	832 (50)	188 (172—204)

**Birth order/birth interval**						

First	193 (16)	202 (184—221)	235 (18)	167 (151—183)	308 (19)	189 (171—207)

2nd or 3rd rank, interval ≤ 2 yrs	136 (11)	202 (186—217)	120 (9)	164 (148—180)	201 (12)	192 (177—207)

2nd or 3rd rank, interval > 2 yrs	180 (15)	177 (163—191)	195 (15)	149 (136—161)	212 (13)	175 (161—190)

4th or higher rank, interval ≤ 2 yrs	420 (35)	174 (162—186)	415 (32)	159 (147—172)	521 (31)	174 (162—187)

4th or higher rank, interval > 2 yrs	262 (22)	215 (200—229)	321 (25)	178 (163—194)	406 (24)	203 (187—219)

**Place of birth**~						

Home	1047 (88)	184 (174—194)	1139(88)	180 (169—190)	1417 (85)	181 (169—192)

Health facility	79 (7)	184 (167—201)	99 (8)	151 (138—165)	246 (15)	179 (161—196)

**Mode of delivery**						

Non-caesarean	1178 (99)	180 (169—191)	1268(98)	179 (169—190)	1646 (99)	180 (169—191)

Caesarean‴	8 (1)	203 (184—223)	9 (1)	152 (138—166)	17 (1)	190 (175—204)

**Delivery assistance**~						

Health professional	95 (8)	182 (164—199)	109 (9)	151 (137—165)	276 (17)	178 (160—197)

Non-health professional^	1019 (86)	188 (176—201)	780 (61)	180 (169—192)	1362 (82)	183 (169—197)


n (%) Weighted number, and proportion of children < 5 years old who died during the under-five period (0–59 months); * All U5MR estimates with 95% confidence interval (CI) were rounded to a whole number; + Wealth was assessed based on household assets (radio, television, fridge, bicycle, motorcycle, car, telephone, electricity, and type of floor material used in rooms) that were consistent across the combined NDHS data sets; ^ Traditional birth attendant, relative or friend; ‴ Caesarean section is a combination of both elective and emergency caesarean; ~ Proportion varies between groups due to missing values; yrs Years.

### Trends in U5M

**Figure 1:**
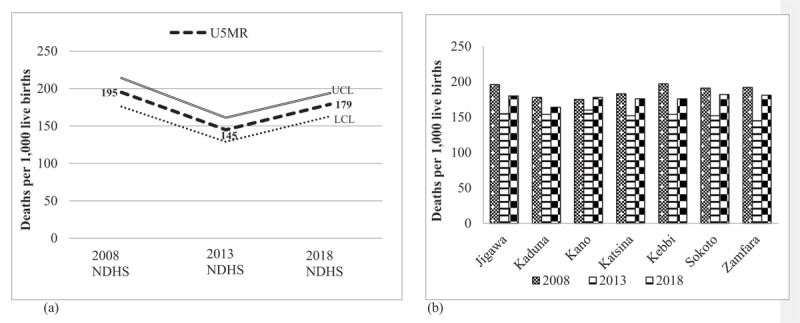
**a** Trends in singleton under-5 mortality rate (U5MR), with 95% confidence interval (lower-class limit [LCL] and upper-class limit [UCL]) in Northwest geopolitical zone (NWGZ) by year of Nigeria Demographic and Health Surveys (NDHS), 2008-2018. **b** Trends in U5MR in NWGZ states by year of NDHS survey.

In NWGZ, a V-shaped trend for U5MR was observed over the 10-year study period, which indicated that U5MR steadily declined by approximately 26% from 195 under-5 deaths per 1,000 live births in 2008 to 145 deaths per 1,000 live births in 2013; however, it dramatically rose by approximately 24% from 145 deaths to 179 deaths per 1,000 live births in 2018. The level of U5MR in NWGZ showed an insignificant decrease over the 10-year study period, averaging only an 8.2% decline over the decade. A similar trend was also noted among the NWGZ states; however, it remains unclear how the improvements in child survival over the five years (2008–2013) were reversed (Figure 1b). Across the seven states, the pace of decrease or increase was uneven and non-significant. Children (<5 years of age) whose mothers could not read had higher U5MR than those whose mothers could read whole or parts of sentences. U5MR disparities existed across the independent study factors (Table 1).

### Factors associated with U5M

As shown in Table 2, children (<5 years of age) born to mothers aged younger than 20 years (aOR = 1.35, 95% CI: 1.08–1.68) or those aged between 40 and 49 years (aOR = 1.20, 95% CI: 1.04–1.40) had a significantly increased odds of mortality compared to those aged 30–39 years. We also noted a significantly higher odds of mortality among children (<5 years of age) whose mothers had no formal education (aOR = 1.53; 95% CI: 1.17–2.01) and those whose mothers had primary education (aOR = 1.80, 95% CI: 1.40–2.33) compared to children of mothers with secondary or higher education. The collinearity assessment in this study revealed that when we swapped maternal educational level with paternal educational level in the final model, children whose fathers had no schooling (aOR = 1.42, 95% CI: 1.23–1.64) or had primary education (aOR = 1.31, 95% CI: 1.10–1.55) were more likely to die before their fifth birthday. An increased likelihood of under-5 deaths was also noted when the maternal educational level was interchanged with maternal literacy in the final model; children whose mothers could not read had higher odds of U5M (aOR = 1.55, 95% CI: 1.29–1.86).

**Table 2:** Under-5 mortality model, and distribution of characteristics reported in three demographic and health surveys in the northwest geopolitical zone, Nigeria, 2008–2018.


VARIABLE	N*	N%Ψ	CRUDE MODEL†§	ADJUSTED MODEL†§

**Year of Survey**				

2008	8529	1096 (29)	Ref	Ref

2013	11442	1181 (32)	0.74 (0.63—0.87)	0.76 (0.65—0.87)

2018	12045	1468 (39)	0.96 (0.84—1.10)	1.00 (0.89—1.13)

**Residence type**				

Urban	7044	580 (15)	Ref	Ref

Rural	24971	3165 (85)	1.56 (1.31—1.86)	1.28 (1.10—1.50)

**North West state**				

Jigawa	4006	505 (14)	Ref	Ref

Kaduna	4872	485 (13)	0.84 (0.64—1.10)	1.00 (0.81—1.25)

Kano	7950	942 (25)	1.00 (0.84—1.19)	1.19 (1.00—1.41)

Katsina	5500	578 (15)	0.85 (0.71—1.01)	0.92 (0.78—1.09)

Kebbi	3054	395 (11)	1.19 (0.97—1.46)	1.21 (0.99—1.47)

Sokoto	3005	385 (10)	1.15 (0.95—1.38)	1.25 (1.03—1.51)

Zamfara	3628	454 (12)	0.93 (0.78—1.12)	1.04 (0.86—1.25)

**Household wealth index**+				

Rich	6662	521 (14)	Ref	

Middle	12789	1589 (42)	1.73 (1.48—2.02)	

Poor	12564	1635 (44)	1.74 (1.47—2.04)	

**Mother’s education**				

Secondary or higher	3791	227 (6)	Ref	Ref

Primary	3942	405 (11)	1.72 (1.32—2.24)	1.53 (1.17—2.01)

No education	24282	3113 (83)	2.21 (174—2.81)	1.80 (1.40—2.33)

**Mother’s literacy level**				

Able to read	5697	393 (11)	Ref	

Cannot read	26229	3330 (89)	1.88 (1.58—2.22)	

**Mother’s age**				

< 20	2085	277 (7)	1.61 (1.34—1.94)	1.35 (1.08—1.68)

20—29	15693	1784 (48)	1.03 (0.93—1.13)	0.99 (0.88—1.12)

30—39	10689	1210 (32)	Ref	Ref

40—49	3548	474 (13)	1.20 (1.04—1.39)	1.20 (1.04—1.40)

**Mother’s body mass index**				

Normal	16733	2010 (73)	Ref	

Underweight	3076	338 (12)	0.89 (0.78—1.02)	

Overweight	2765	294 (11)	0.84 (0.70—1.00)	

Obese	1102	115 (4)	0.73 (0.54—1.00)	

**Wanted pregnancy**				

Wanted then	30424	3494 (96)	Ref	

Wanted later	898	97 (3)	0.95 (0.72—1.25)	

Wanted no more	284	31 (1)	0.88 (0.55—1.41)	

**Father’s education**				

No education	18943	2464 (68)	1.67 (1.45—1.92)	

Primary	4552	519 (14)	1.48 (1.25—1.74)	

Secondary or higher	7584	624 (17)	Ref	

**Mother’s perceived baby size**				

Average or larger	26469	2855 (79)	Ref	Ref

Small or very small	5031	753 (21)	1.46 (1.30—1.65)	1.43 (1.28—1.61)

**Sex**				

Female	15876	1769 (47)	Ref	

Male	16139	1976 (53)	1.10 (1.01—1.21)	

**Birth order/birth interval**				

First	5257	719 (19)	1.57 (1.37—1.81)	1.47 (1.27—1.71)

2nd or 3rd rank, interval ≤ 2yrs	2657	441 (12)	1.90 (1.57—2.30)	1.90 (1.58—2.28)

2nd or 3rd rank, interval > 2 yrs	6470	561 (15)	Ref	Ref

4th or higher rank, interval ≤ 2yrs	12755	1158 (31)	1.12 (0.99—1.27)	1.05 (0.90—1.22)

4th or higher rank, interval > 2 yrs	4877	866 (23)	1.98 (1.72—2.29)	1.86 (1.58—2.19)

**Contraceptive use**				

**Yes**	1525	103 (3)	Ref	

**No**	30490	3642 (97)	2.07 (1.59— 2.71)	

**Place of birth**				

Home	27980	3311 (91)	1.42 (1.18—1.72)	

Health facility	3801	337 (9)	Ref	

**Mode of delivery**‡				

Non-caesarean	31762	3701 (99)	Ref	

Caesarean‴	170	27 (1)	1.31 (0.63—2.72)	

**Delivery assistance**				

Health professional	4290	376 (11)	Ref	

Non-health professional^	24398	2911 (89)	1.39 (1.17—1.66)	


* Combined total weighted live births (singleton) across each of the variable groups between 2008 and 2018; Ψ Weighted number, and proportion of children < 5 years old who died during the under-five period (0–59 months); † logistic regression model was used to estimate the odds ratio with 95% confidence interval; § Missing values were not included in the model; ‡ Mode of birth was excluded from the adjusted model because only 1% had caesarean section during the study period; ‴ Caesarean section is a combination of both elective and emergency caesarean; ^ Traditional birth attendant, relative or friend; Ref Reference category; yrs Years; + Wealth was assessed based on household assets (radio, television, fridge, bicycle, motorcycle, car, telephone, electricity, and type of floor material used in rooms) that was consistent across the combined NDHS data sets.

Children born to mothers residing in rural areas (aOR = 1.28, 95% CI: 1.10–1.50), as well as those whose mothers did not use any form of contraception (aOR = 1.47, 95% CI: 1.12–1.94), had a higher probability of U5M. A similar outcome was equally observed when the household wealth index substituted the residence type in the final model; children of mothers from poor households (aOR = 1.40, 95% CI: 1.19–1.64) or middle-income households (aOR = 1.32, 95% CI: 1.11–1.57) had increased odds of U5M. Other factors that showed a significantly increased likelihood of deaths among children (<5 years of age) included children whose body size after birth was perceived as small or smaller, second- or third-order born children with shorter birth intervals (≤2 years) and fourth- or higher-order born children with a longer interval (>2 years) (Table 2).

## Discussion

In NWGZ, a modest decrease in U5MR was recorded between 2008 and 2013; however, this gain diminished as U5MR ascended from 2013 to 2018. A similar trend pattern was observed among its seven states. The overall average U5MR for NWGZ was 171 deaths per 1,000 live births, which stands well above the most recently reported national average (132 deaths per 1,000 live births) [5]. An uneven variation of U5MRs within the NWGZ states was observed, and these differentials are attributable to socioeconomic inequality and inadequate access to skilled health personnel and health facilities. U5MR remains very high in NWGZ and requires further improvement and urgent attention to actualise the Sustainable Development Goal target (25 under-5 deaths per 1,000 live births by 2030). Further findings showed that contraceptive use (non-use), baby size at birth (small or very small), birth order (second to fourth or higher) with a shorter birth interval (≤2 years), maternal age, residence type (rural), and maternal education (no formal or primary education) were significantly associated with U5M in NWGZ.

Non-use of any form of contraception has been previously documented to increase the risk of U5M in Kenya [21], Bangladesh [19], and Nepal [22]. In this study, we observed that mothers who did not use any form of contraception were significantly more likely to experience U5M compared with those who used contraception. This outcome is not unexpected because in NWGZ, only 6.7% of sexually active unmarried and married women aged 15–49 years old used any form of contraception between 2013 and 2018 [5]. The side-effects (e.g. womb damage, menstrual irregularities, delay in return to fertility, partner objection and difficulties in breastfeeding) [23,24] arising from contraceptive use can be linked to the increased likelihood of U5M noted among NWGZ women of reproductive age. Additionally, cultural beliefs or religious practices might have played a key role in the increased odds of under-5 deaths among mothers who did not use contraception. It has been previously reported that women of reproductive age who practice Islam are less likely to use any form of contraception [25,26]. This finding sturdily supports the need for effective public health interventions (e.g. intensifying media campaigns on adverse effects of high-risk births, providing free family planning essentials at primary health facilities and educating women and their partners/husbands on the benefits of contraceptive use) to increase the use of contraception in scaling down U5M.

Average or larger-sized children, as perceived after birth by their mothers, had a lower odd of U5M than those perceived as small- or smaller-sized. This finding is in contrast with that obtained by Yaya et al. [14]; however, a range of similar studies indicated a significant relationship between the two factors [16,20,27,28,29]. The approach mothers used in measuring their infant’s size after birth remains unclear. Thus, caution should be exercised in concluding this finding, as preterm and small gestational age infants were not classified in the 2018 NDHS. A previous study conducted in Bangladesh indicated that approximately 75% of deaths related to low birth weight in children were linked to a preterm condition [30].

Second or higher birth orders with shorter birth intervals (≤2 years) significantly increased the odds of U5M in NWGZ. A similar significant relation was reported in Kenya [31], Bangladesh [19], and Tanzania [27]. However, our current outcome contradicts a study conducted in Benin, which revealed that fourth- or higher-order births with >2 years of birth had a significantly increased likelihood of U5M. Our finding can be validated by the insufficient economic resources, particularly in low-socioeconomic status households, resulting in competition among siblings. This adversely impacts maternal health and well-being [32]. Inadequate care and attention given to higher birth-order children might be another contributing factor [32]. This finding illustrates the need for public health interventions to promote and scale up the importance of birth spacing, particularly in low-socioeconomic status communities.

An increased likelihood of under-5 deaths was noted in younger mothers aged below 20 and older mothers aged above 40. These findings are not consistent with the maternal age differentials in U5M previously documented in Bangladesh [19]. This finding can be attributed to physical immaturity and inexperience regarding child-rearing, particularly young motherhood. Meanwhile, medical factors (e.g. gestational diabetes, hypertension, operative vaginal delivery and antepartum haemorrhage) [33,34], which were earlier detailed to be associated with older mothers, might be linked to the significantly increased U5M related to older NWGZ mothers. Public health interventions targeting younger mothers to delay their first pregnancy due to its associated adverse effect (e.g. obstetric fistula) and educating older mothers to seek timely appropriate medical care and check-up during pregnancy remain crucial, leading to a reduction in U5M in NWGZ.

These findings reaffirm a significant relationship between poor household economic status and U5M in NWGZ, which is attributable to a recent report that approximately 87% of the entire poor population in Nigeria resides in northern Nigeria [35]. The poverty constraint affects mothers in several ways, such as gaining access to modern health facilities for maternal healthcare services, non-polluted fuel and a socially developed environment with good water and sanitation infrastructure. It also impacts mothers’ health-seeking behaviour, which often leads to the increased probability of U5M. Therefore, it is vital and urgent for both the local and state governments to revitalise their poverty intervention initiatives, especially in conflict-infected communities. Similar to previous findings from studies conducted in Bangladesh [36], Chad [37], and Tanzania [27], this study also indicates that children whose mothers have primary education or no formal education exhibit significantly increased odds of U5M compared to those who have secondary or higher education. However, a recent study in Kenya [38] is not in agreement with our findings, which reported an insignificant association between a mother’s education level and U5M. Our findings might be related to an uneducated mother’s inability to understand the benefits of good childcare practices, such as hygienic behaviours, immunization, preventative care and suitable and timely feeding. Moreover, uneducated mothers are more likely to adhere to sociocultural practices that negatively affect child survival [39].

The following are the limitations of this study: (1) reasonable numbers of births and deaths could have been misrepresented, as only surviving mothers participated in the surveys; (2) maternal health status (e.g. infection, diabetes and hypertension) before or during childbirth was not incorporated in the study analysis as it was not documented in the 2018 NDHS, which might have affected our estimates. Moreover, postpartum depression, previously reported to be significantly related to U5M in Taiwan [40], was not considered due to the absence of data; (3) data on the medical health condition and causes of death (e.g. birth asphyxia, jaundice and sepsis) of children aged below five years were not available for examination; (4) the assessed factors, such as the perceived size of the child’s body at birth by mothers, might have impacted our estimates, as the rationale or criteria applied remains unclear.

This study has the following strengths. (1) Births and deaths in the study were limited to a five-year period to reduce recall bias of dates of birth and death and possible changes in household factors (e.g. economic status); (2) this was a population-based study, with more than 90% of the same ethnic group practicing the same culture and religion, as well as the same economic livelihood and social lifestyles, which increased the validity of our estimates; (3) this study highlighted specific evidence on NWGZ key factors associated with U5M, which would inform the targeted intervention initiatives to scale down the number of under-5 deaths.

## Conclusion

The findings obtained from the examined factors related to U5M in NWGZ showed that contraceptive use (non-use), baby size at birth (small or very small), birth order (second to fourth or higher) with a shorter birth interval (≤2 years), maternal age (<20 years or ≥40 years old) and maternal education (no formal or primary education) reported a statistically significantly increased likelihood of U5M. This outcome indicates urgent interventions for child survival at both community and individual levels, which involve educating mothers on the benefits of contraceptive use, child spacing, kangaroo mother care of small-sized babies, and promoting regular medical check-ups for older mothers. To substantially scale down U5M in NWGZ, these interventions should primarily aim at mothers belonging to low-socioeconomic status households.
